# Enacting Gender: An Enactive-Ecological Account of Gender and Its Fluidity

**DOI:** 10.3389/fpsyg.2022.772287

**Published:** 2022-05-09

**Authors:** Mahault Albarracin, Pierre Poirier

**Affiliations:** ^1^Department of Computer Science, Université du Québec à Montréal, Montréal, QC, Canada; ^2^Institut de Recherche et d'Études Féministes, Université du Québec à Montréal, Montréal, QC, Canada; ^3^Department of Philosophy, Université du Québec à Montréal, Montréal, QC, Canada; ^4^Institut des Sciences Cognitives, Université du Québec à Montréal, Montréal, QC, Canada

**Keywords:** gender, gender fluidity, cultural affordance, ecological psychology, enaction, active inference, social scripts

## Abstract

This paper aims to show that genders are enacted, by providing an account of how an individual can be said to enact a gender and explaining how, consequently, genders can be fluid. On the enactive-ecological view we defend, individuals first and foremost perceive the world as fields of affordances, that is, structured sets of action possibilities. Fields of natural affordances offer action possibilities because of the natural properties of organisms and environments. Handles offer graspability to humans because of physical-structural properties of handles and the anatomical-physiological properties of humans. Although humans live in fields of bodily, action, and cultural affordances, our work focuses on cultural affordances, where action possibilities are offered to individuals because of the normative responses of individuals in that culture. Knocking on a door affords entrance because knocking provides cultured individuals on the other side of the door an affordance to which they themselves behave normatively. Usually, behaving normatively in response to cultural affordances brings about sequences of perception-action loops, which we will call “scripts”: for instance, closed doors afford knocking, which affords the individual inside opening the door, which affords an interpersonal meeting, which (may) afford entrance. Although the notion of script has a strong cognitivist flavor, one of the aims of the paper to provide an ecological account of scripts, to show that what cognitivists viewed as representations (or representational structures) are in fact environmentally structured perception-action loops. On our account of gender, gendered cultures build and maintain gendered cultural affordance landscapes, that is, landscapes in which the action possibilities individuals face are normed according to a specific body type or situation; most often (assigned) biological sex. Individuals enact a given gender when they come to perceive the affordances reserved for one gender by their culture and respond in the culturally normative way, thus enacting gendered sequences of perception-action loops (i.e., gendered scripts). With the shifting landscapes of cultural affordances brought about by several recent social, technological, and epistemic developments in some cultures, the gendered landscapes of affordances offered to individuals in these cultures have become more varied and less rigid, thus increasing the variety and flexibility of scripts individuals can enact. This entails that individuals in such cultures have an increased possibility for gender fluidity, which may in part explain the increasing number of people currently identifying outside the binary.

## Introduction

We propose to understand cultural social scripts in an enactive-ecological framework, which integrates the enactive idea that cognitive capacities are enacted through dynamical loops with an environment with the ecological ideal that the environment should be conceived as a field of affordances. Under this view, script-based interpersonal negotiation can be linked to the concept of a cultural niche, understood as a landscape of cultural affordances (Ramstead et al., 2016). As we will see, cultural affordances are possibilities for action whose perception, based both on multisensorial biological (homeostatic, bodily, skilled capacities, etc.) and cultural cues (practices, hermeneutic resources, norms, etc.), constrain which actions are pre-reflectively felt possible by an individual in a given culture. Although cultural affordances are not mere conventions, the conventions established, and sometimes enforced, by a culture may be part of the causal setup responsible for the presence of the affordance. It is a mere convention that the color red has been chosen to signal the obligation to stop at an intersection. But once all drivers have internalized the relation between the red light and obligation to stop, in such a way that they will automatically stop once the traffic light (in the proper relation to the direction in which their car is moving) turns red, then the red traffic light, in that culture, affords stopping. To be sure, the causal relationships that sustain this affordance are more tortuous than if giant doors were used to stop traffic at intersections, but both the giant doors and the red light still afford stopping. In an industrialized urban setting, thus, most people pre-reflectively feel that dark rooms can be lit (a switch somewhere affords lighting), fire-fighters can be called should there be a fire emergency (phones afford calling 911—note here too that he number chosen is mere convention but not the relationship between calling and the presence of fire-fighter in a culture that provides the service) and social interactions with strangers can be cut-off at will given a few polite niceties (polite conversation affords control over social interactions). Things may go wrong, to be sure: the switch may be broken, the 911 service may be offline or someone may fail to recognize, or ignore the polite niceties of conversation. Following Gallagher and Zahavi (2008), we understand pre-reflective consciousness as an implicit and first-order, as opposed to explicit (e.g., conceptual) and higher-order (e.g., thought about thought; Rosenthal, 1986), awareness we have (e.g., of possible actions) before we reflect on our experience. Merleau-Ponty famously illustrated the nature of pre-reflective consciousness with his analysis of football (soccer): “The field itself is not given to him, but present as the immanent term of his practical intentions; the player becomes one with it and feels the direction of the “goal,” for example, just as immediately as the vertical and the horizontal planes of his own body” (Merleau-Ponty, 1942: p. 169). The fact that possibilities for actions are first and foremost felt pre-reflectively does not mean that they can, on occasion, be the object of reflective thought. If I witness an accident, I will feel that my phone affords calling 911, but might reflectively decide against acting upon this opportunity if I remember that its battery is dead. Thus, the fact that affordances are felt pre-reflectively does not mean also that the actions undertaken and their reasons may not be reflected upon on occasion or if the situation demands it. Nevertheless, this is how, ecologically, the industrialized urban setting is pre-reflectively felt by most. As they act in a given situation, individuals enact a subset of their landscape of affordances as the situation's field of affordance (Bruineberg and Rietveld, 2014; Kirchhoff and Kiverstein, 2019). A situation's field of affordance is the pre-reflexively felt structure of possible actions in that situation. No two agents enact the same field of affordances in any given situation, since fields of affordances depend on multisensorial biological and cultural cues, and no two agents have identical bodies (homeostatically, kinesthetically, etc.) and no two cultures (which can be as finely individuated as the individual) offer identical norms and practices. Since humans share biological and cultural commonalities at various spatial and temporal scales, however, their collective fields of affordances in a given situation thus form a space, in which there is structure at multiple temporal and spatial scales. We call this structure, which will be important to understand the possibility and constraint on gender fluidity, “the fields-of-affordances space.” Any action undertaken by a specific human agent in a given situation will bring forth a new field of affordances, belonging to a new fields-of-affordances space, opening new action possibilities, and so on. Scripts, as we will argue, are biologically and culturally constrained sequences of such perception-action loops, and gender, while not entirely reducible to scripts, has its own subset of scripts. Specifically, they are the perception-action loops constrained by the individual's culturally shaped biological body, the way culturally-shaped biological bodies are perceived by others, the cultural norms that shape bodies, brains and behaviors, and the political forces that shape those norms. This, unlike neuroreductionist views, entails that any individual that enters new situations has a possibility for gender fluidity, that is, of enacting new gendered affordance-actions loops (we expand on the notion of gender fluidity below). These possibilities reflect the structure in the fields-of-affordance space: individuality but also commonalities at multiple temporal and spatial scales.

With the shifting cultural norms and embodiment possibilities brought about by a number of contemporary political, social, technological and epistemic developments, the norms of gender are becoming less rigid (Amato and Booth, 1995; Scott et al., 1996; Brooks and Bolzendahl, 2004; Knight and Brinton, 2017), which translates into an increase in the variety of scripts agents can negotiate and a concomitant decrease in strength of the normative constraints put on individuals as they move from situation to situation (Tallichet and Willits, 1986; Bryant, 2003), as evidenced by the increasing number of individuals identifying outside the binary. The affordance negotiation approach allows us a critical stance on the cognitive neuroscience of gender.

## An Enactive-Ecological Framework

The enactive-ecological framework we adopt is here closely related to the Skilled Intentionality Framework (SIF) developed by Bruineberg, Rietveld and Kiverstein in a number of papers Bruineberg et al. (2021); Kiverstein and Rietveld (2021), which builds on the enactive tradition in cognitive science initiated by Varela et al. (1991b), on Gibson's (1979) ecological approach to perception, and on the more recent Free Energy framework developed by Friston (2019). This framework, we believe, offers the conceptual apparatus necessary to revisit Gagnon's notion of sexual scripts (Simon and Gagnon, 1986), and its conceptualization of gender, giving us a notion of gender that has the explanatory power to understand fluidity. The proper way to integrate important features from these different research traditions is currently under debate, and there are those who think that it will prove impossible to integrate them (or one of them with the other two) for they are ultimately incompatible (REFS). This paper is grounded on the possibility that they can be in something like the manner Bruineberg and colleagues propose. It should be mentioned upfront, however that we go further than them in accepting the brain mechanisms proposed by Friston (2005) provided (1) that strict adherence to the personal/subpersonal distinction (Dennett, 2002) be maintained and, for that reason, that we refrain from describing the internal mechanisms in intentional, the way like Orlandi (REFS) does. We briefly present the Framework, thus understood, below.

Enactivism views cognitive properties on a continuum with biological properties, the properties that give rise to and maintain life. By anchoring cognition to life, enactivism adopts an inherently embodied conception of cognition. The concept of embodiment also has a long tradition in feminist thought (for an overview, see Threadcraft, 2016) and although the two concepts are related in many ways, their exact relation still has to be ascertained (Pitts-Taylor, 2013). On Varela et al. (1991a) account, life is understood as the self-organization and homeostatic maintenance of chemical reactions, a combination they called autopoiesis. The most basic way for a self-organized autocatalytic set of chemical reactions to achieve homeostasis is to build and maintain a semi-permeable membrane to enclose itself in, making it able to weather punctual changes in its milieu. By doing so, the autocatalytic set of reactions takes its first step in the journey of variational free-energy minimization: it makes viable internal states more probable than states less conducive to self-preservation. For a living organism, variational free-energy is an information-theoretic quantity that is an upper bound on the surprisal of a set of states sampled from its (internal or external) environment, given a generative model of the causes of the sample. The Free-Energy Principle (Friston et al., 2006) states that any adaptive living organism resists disorganization by minimizing its free-energy. All adaptive living organisms are thus posited to possess a generative model of their environment.

A more sophisticated way for an organism to preserve homeostasis and minimize surprise is to be sensitive to changes in conditions inside and outside its membrane, and to react appropriately to these changes, notably by sensing and reacting to these changes conditions, creating dynamical perception-action loops between organism and internal or external) environment (Cisek, 2019). Active maintenance of homeostasis by action perception loops gives homeostatic value to the environment: some of its states afford the organism motor actions that preserve homeostasis, and some don't. It gives a basic kind of meaning to the world (Thompson, 2006; Di Paolo et al., 2018): (1) perceptual and action capabilities are evolved to detect and react to those conditions in the world that are beneficial for maintaining self-organization, and to those conditions that are detrimental to it; (2) and the organism will be attracted to the conditions that are beneficial and repulsed by those that are not.

In ecological psychology (Gibson, 1979), affordances are properties or features of objects (situations, states, events, etc.) that afford actions to an organism. Affordances are possibilities for action that the organisms' environment supplies (affords) to individuals whose embodied characteristics allow them to can perform the afforded action. Warren (1984) for instance climbability as it relates to embodied characteristics of individuals, finding a direct relation between these characteristics and individuals' perception of climabability. Van der Meer (1997) empirically studied affordances for movement in different groups of children and adults, measuring their tendencies to duck under an obstacle given their embodied characteristics. Affordances depend on sets on body properties and skills and sets of environmental features, and are as such persistent relational features of the environment. Individuals organisms are able to perform actions to the extent they possess the necessary body configuration, physiology, and skills. Since body configuration, physiology and skills change throughout the organism's lifespan, its landscape of affordances, the affordances it finds in a given environment, change as well. For a given organism, the set of affordances supplied by its environment at a given time is determined by the content of its (local, global) environment at that time, as well as its body configuration, physiology and skills at that time. The set of affordances an individual (or type of) organism has at a given point in time are called the individual's ecological niche (Gibson, 1979). The space of affordances currently available to an organism is called the organism's affordance landscape (Bruineberg and Rietveld, 2014). To an organism able to perceive them, affordances meaningfully structure the environment, making it positively value certain aspects of the environment (e.g., nourishment affordances) and negatively value others (e.g., hurting affordances). In the context of enactive-ecological psychology, perception-action loops become actions-affordance loops: given an affordance landscape, the free-energy-minimizing organism will opt for the action that most minimizes free-energy, which will bring about a new affordance landscape, a new action free-energy-minimizing action, and a new affordance landscape, and so on. de Haan (2020) distinguishes various dimensions of fields of affordance that will prove useful for our model: (1) width (how large is the range of action possibilities perceived), (2) depth (how long is the sequence of actions one is pre-reflectively aware of) and finally (3) height (how salient is the action possibility in the field of affordance. For instance, if there is an apple in my immediate environment (an orchard, say), I will perceive the possibilities of nourishment and throwing, but perhaps not much else (relatively narrow field). I will also feel pre-reflectively that eating the apple will involve a long sequence of biting, chewing and swallowing, whereas throwing the apple will feel like a short duration affair. Finally, if I am hungry, the nourishment affordance in my field will have a high salience whereas the throwing affordance will have a low salience. But if a bear shows up in the orchard the salience of the affordances will probably switch.

Homeostasis, as described so far, is a reactive process (Ashby, 1962), and so are the affordance loops that help maintain it. Following Cisek (2019: p. 6), we take it that “the evolutionary history of the nervous system is essentially a history of the continuous extension of such control further and further into the world.” Brains allow organisms predictive homeostasis, or what Barrett (Barrett et al., 2016), following Sterling (Sterling, 2012), calls allostasis. Instead of passively waiting for internal or external conditions to change, allostatic organisms try to anticipate the coming changes in order to respond preemptively to insure homeostasis. One of the most influential recent advancements in neuroscience is the development of a framework, now often referred to as the Active Inference Framework. This framework understands the brain as the predictive system (Friston et al., 2006) that serves as the mediating organ between organism, body and environment (Fuchs REF) responsible for the anticipatory abilities of organisms. The computational mechanism grounding the Active inference Framework has grown in detail and complexity over the past 15 years, but we will focus here on the details that are relevant for our purpose, presenting them as they prove relevant. The computational mechanism is set up as a hierarchy layers where neurons at one hierarchical level are tuned by learning mechanisms to generate the signals they are about to receive from neurons one level below.

Described using machine learning concepts, each layer encodes a set of generative models in a superposed fashion, each of which could be used at a point in time to generate the upcoming signal. The notion of model is controversial in both the enactive and ecological communities, but nothing more should be inferred by this use, taken from machine learning (see e.g., Salakhutdinov and Hinton, 2012), than that neurons at one hierarchical come to be able to generate the activity they are about to get from the layer one level below. On a short timescale, the upward flow of so-called “prediction errors,” that is, signals generated at each layer by the difference between activity generated within the layer and activity received from the layer belowserves model selection, that is, selecting which generative model will be active in a given layer. On a longer timescale, the upward flow of signals serves learning; that is, changing the connection weights between layers and thus changing the models encoded in a layer. This allows the brain to minimize the amount of discrepancy between generated and received activity, which, under certain assumptions, minimizes its free energy. The brain can also minimize this quantity by changing the organism's internal and external environment through the action of the its effectors, a process called “active inference.” If the environment is viewed as a model of the organism, then active inference can also be understood as model selection (Constant et al., 2019). The environment can also be changed at different timescales. At the shortest of timescales, any movement from the organism changes what is sampled from the environment, without the environment changing itself; at increasingly longer scales, objects can be moved, structures can be built, dynamics set in motion, institutions established, cultures evolved.

The hierarchical nature of the architecture imposes an order to the system's signal generating activity. Neurons at the sensory periphery receive very local and short timescale signals and this is what they will attempt to generate; similarly for motor neurons: they generate the local and short timescale signals that activate muscle fibers. Neurons at the next level up receive signals from the layer below. Initially, when the layer below has no working generative model, the discrepancy between the signals it generates and those it receives is large and that second layer gets to learn a model of the activity of the first layer. As the first layer improves at generating incoming signals, however, it sends less and less signal upward, except in novel environments and situations. The result of this arrangement is that higher and higher layers encode the probability distributions of properties at larger and larger spatial scales, longer and longer timescales; properties that are more and more abstract with respect to the raw sensory signals.

The move from low level to high level in the hierarchy also sees a progressive integration of signal from multiple sensory (interoceptive, proprioceptive and exteroceptive) and effector (motor, endocrine). As signals from multiple sensory and effector hierarchies are integrated at generative models embody the affordances of objects and situations: neurons attempt to generate the signal which, given context signals from levels above and prediction error signals from below, would (predictively) best maintain homeostasis if it were sent down the hierarchy (and will receive an interoceptive feedback signal that contains information about actual maintenance of homeostasis). Generative models at higher levels thus encode the probability distribution of affordances.

And because of the hierarchical nature of the generative models, this means these probability distributions of affordances are arranged in a nested hierarchy: restaurants afford many actions (eating, spending money, sitting, meeting people, going to the restroom, etc.) that themselves afford many actions. In the case of humans, many of these affordances are cultural: actions afforded by a culturally designed objects (e.g., a light switch), practices (e.g., the multiplication algorithm) or institutions (e.g., education): the light switch affords lighting dark rooms, the multiplication algorithm affords multiplying any two numbers, and education affords, e.g., working in certain fields. Affordances are cultural when it is human culture that designed its objects, practices and situations and sustains the possibilities for action. It is human culture that designed the situation in which a switch on a wall affords illuminating the room when it is dark and sustains the switch's affordance (by enabling the processes that keep electricity coming to the switch and bulb, ensure the manufacture of bulbs, etc.). The existence of cultural affordance opens the possibility for the presence of sensorimotor cultural-affordance loops: sensorimotor affordance loops where it is culture that sets up and sustains the artifacts processes such that some objects or situations afford actions.

## Cultural Scripts in Ecological-Enactivist Framework

A script is a form of knowledge structure about the sequence of events to be produced in a given setting. A typical example is the restaurant script (Schank and Abelson, 2013). Upon entering a traditional restaurant, one expects to be seated, met by a person assigned to wait on her, offered a menu, and so on. This knowledge is thought to be encoded in a memory structure that develops as individuals are exposed to multiple varied instances of the setting. An important point to note about scripts is that they are normative sequences of perceptions and actions (on the normative nature of affordances, see Heras-Escribano, 2019). Upon opening the door, most restaurants will offer a familiar field of affordance (multiple tables and chairs, dining customers, waiting staff), perception of which should prompt someone who intends to eat at the restaurant to stop and wait to be met by a member of the waiting staff. By behaving in a manner that respects the norms of the restaurant script, the individual plays the “customer role.” Employees and delivery persons, etc., given a similar perception upon entering a restaurant, will not behave in a manner that respects specific norms of the restaurant script, and thus will play the customer role.

This typical example illustrates a cultural script, because it is embedded in a culture that creates the objects and situations that make up the script and prescribes (sometimes enforces) the norms individuals must follow to play a role in the script (Goddard and Wierzbicka, 2004; Goddard, 2009; Haley and Haley, 2016; Constant et al., 2019). This does not mean that all scripts are cultural. Natural scripts are set up by the organism's need to maintain its precarious self-organization in its natural environment. Although these may be quite basic (e.g., bacteria chemical gradient ascent or descent scripts), they may also be quite complex (e.g., the prey-predator scripts). Though the roles in such scripts may be learned, following their norms is rarely a matter of choice. Although natural scripts are interesting in their own right, and we should never underestimate their presence in human cognition, we shall focus here on cultural scripts, because gender is, we shall claim, a cultural script. But before we get to gender, we will argue presently that cultural scripts are better understood within the enactive-ecological framework introduced above.

In what follows, we shall explain how cultural scripts can be viewed as sensorimotor cultural-affordance loops and then show the explanatory power thereby gained by the concept of cultural script by explaining, in the next section, the fluidity of cultural script. Cognitive science traditionally construes scripts as internally memorized sequences of actions (Schank and Abelson, 1977), which is a representationally and computationally costly, and sometimes brittle, way of structuring behavior. Enactive-ecological cognitive science offloads some of those representations and computations to the environment. One does not learn a script by acquiring a representation of the script but by becoming sensitive to the fields of affordances an environment presents and skillful at producing the actions thereby afforded. On the active inference view adopted here, one becomes sensitive to an affordance in a field when one's generative models predict the affordance when it is about to become present (e.g., they predict that by opening the fridge's door, nourishment will be afforded) and prepares her whole organism to act upon the possibility offered by the object or situation (Fuchs, 2017). Acting upon the action possibilities offered by the environment normally changes the field of affordances, either because its laws, structures or mechanisms make it so or because other humans who will normally respond in a culturally normed fashion to the field. This new field of affordances may lead to new actions, and thus new fields of affordances, and so on. Upon entering a restaurant, one enters into a culturally constructed environment that affords eating, but is first met however with a setting whose norms culturally afford waiting. Violating this norm might bring a response from the restaurant's staff or owner that affords exiting the restaurant. Waiting, on the other hand, will present staff with a situation that affords offering a seat at a free and clean table as well as a menu to anyone that waits (obviously, this could be described in much more detail). Being seated at a clean table with a menu affords consulting the menu to choose an item, and so on. One progressively masters the script as her brain's higher-level generative models predict affordances and can skilfully execute the actions normatively prescribed. Of course, one can refuse to execute the normative action prescribed by the script, in which case every participant will need to bring conscious thought into pursuance of the social interaction.

With time and experience, one may come to perceive deeper and wider fields of affordances. I may come to perceive the restaurant's entrance as both affording waiting or walking directly to the cash register or to the restroom. Fields of affordances thus become polysemic: the restaurant's entrance comes to mean various things, it directs toward various action possibilities. I may also be pre-reflectively aware that nourishment will be shortly available and that I will be asked to part with some of my money. Larger spatial and temporal events associated become nested as shallow and narrow fields of affordances (e.g., a restaurant's entrance) are nested into deeper and wider ones (e.g., restaurant dining). At that point, one possesses multilevel, polysemic affordance fields. Cultural scripts can be thus seen as sequences of meaningful cultural-affordance loops. Scripts are not in the head of individuals but are distributed between the (enculturated) individual and the cultural niches they inhabit. They are hybrid structures composed of mind and environment, the mind supplying the capacity to perceive cultural affordances and the skills to perform the offered actions, and the environment supplying fields of cultural affordances and, importantly, given the actions normatively performed by the individual, sequences of fields of cultural affordances.

Many cultural scripts are social, that is, scripts an individual has constructed by living in society, and having to exploit its group dynamics. In humans, and many animals as well, the actions of individuals afford actions to other individuals. We saw this already in the restaurant case. The restaurant's entrance affords waiting to be seated, but my waiting affords the waiting staff directing me to an empty table. Groups afford different action possibilities to individuals who perform actions who conform better to their norms. Resources are also afforded to people who conform to the group norms. An example of this can be found in urban cultures through informal giving practices. An individual is never forced to acquiesce to a request from a member of the group, but refusing such a request may mean that one is then cut off from the community should they need help in turn (Hornsey et al., 2003; Griskevicius et al., 2006). Access to resources is thus conditional on the capacity of the individual to conform to group norms [or habitus, Bourdieu (1990a,b)], but is not sufficient. Certain group dynamics afford different things to different statuses of their members, regardless of their conformity to group norms. Certain groups thus predefine a member status which grants more power, and thus more affordances over some resources than others.

Finally, cultural scripts are fluid: new scripts can quickly replace old ones and scripts can form new branches. We explained above that classical computational cognitive science construed scripts as internally memorized sequences of actions. It presumed that representations can be added at little computational cost (as when one writes information on a computer's harddrive). But neuroscience has shown that acquiring new representations is a long process that involves ultimately the slow coordinate change of synaptic weights. Apart from being representationally and computationally costly then, this way of structuring action makes cultural scripts rigid and difficult to change. On this view, the introduction of the fast-food restaurant, for instance, would have required the slow acquisition of new sequences of action, which would have required many trips to fast-food restaurants and the frustration of many sub-optimal experiences in such restaurants. The proposed view better accounts for the cultural fluidity of scripts we observe. There are two sources to this fluidity: the polysemy of fields of affordances, and the fact that fields of affordances can be quickly changed culturally (much faster than reprogramming neurologically encoded sequences of action—older readers can attest that the evolution of texting has outpaced their ability to learn to thumb-type). We saw above that many fields of affordances, and hence the scripts that they constitute, are polysemic; they offer various possibilities for action. Which action will be executed depends on which affordance in the field is higher (has more salience) at a given time. Hence changing a script can simply mean making one affordance in a field more salient than before. This can be brought about learning to focus attention on one aspect of the environment, or saccading more often to it, as opposed to another. Upon entering a restaurant, one has learned to pay attention to many specific features of the field of affordances and can fluidly opt for a number of restaurant-related scripts (eating-in, take-out, using the restroom). Moreover, fields of affordances can be culturally changed, sometimes simply by changing the relative saliency of different features in the environment. The traditional restaurant script has sprouted a number of variants in the last 50 years: the fast-food restaurant, the drive-in, the drive-through, the pick-up, the delivery, etc. On the view we defend, the introduction of fast-food restaurants, the physical structure themselves, introduced the new sequence of actions. Fast-food restaurants, like all cultural settings, provide the sequence and all one has to learn is to become sensitive to the new affordances in an already known field of affordances. Fast-food restaurants introduced a new affordance in a familiar field, indicated, e.g., by the gaudy appearance of fast-food restaurants. Once one becomes sensitive to the fact that the specific appearance of this type of restaurant affords walking up to a service counter, one can easily switch between waiting and walking (to the counter). In time, with cultural evolution, the inside appearance of restaurants became clearly distinguished: posh appearance came to afford waiting to be seated, and gaudy appearance came to afford walking to the service counter. On this view, the fact that most fast-food restaurants have a distinctive appearance is not incidental, but a very part of the cultural script now associated with restaurants.

The rest of the fast-food restaurant script is made-up of affordance-action pairs the individuals already had mastered, either as part of other scripts (from shopping at stores generally, eating in school cafeterias, etc.) or as part of the previous restaurant script. On the enactive-ecological account, cultural scripts are loosely coupled structures that can be decoupled from their original script and recoupled into other scripts as the environment demands. This is a feature we will call their porosity. This can be done because mastery of sensorimotor affordance loops sits at a lower hierarchical level that learns faster than the higher levels where the full temporal and spatial dimensions of the script are integrated. As a result, cultural scripts are much more fluid and quickly responsive to environmental change than natural scripts, such as those evolved by natural selection propounded by evolutionary psychologists such as Baron-Cohen., and as we saw with the introduction of fast-food restaurants, it also makes it much easier to introduce new cultural scripts, Thus enabling cultural creativity. We now see how all of this can be used to understand gender and fluidity.

## The Fluidity of the Gender Script

To illustrate the fluidity of cultural scripts, we can use the gender script as an example. Most cultures produce and maintain a gender script, and it has large scale ramifications on social processes. Power structures have gendered ramifications, and institutions are fashioned according to gendered norms and prescriptions. Social practices idealize certain bodies over others. While the gender script seems stable over time, it has known some notable changes both in the recent and distant past that were well-documented through narrative and iconic records. It is important to note that gender has not been seen as a binary all over the world. Communities have had non-binary gender identifications, from the mexican muxes, the hawaian mahu, the native two-spirit or the Indian Hijras (Picq and Tikuna, 2019). But these cultures have been besieged by western imperialism, which harbored more clearly delineated binaries. Here, again, power relations are crucial. The larger group, taking power through often violent means, imposed a structuring binary perspective. While these cultures have not necessarily lost their semantics or presence, access to institutional resources was and still is strongly dependant on adoption of the controlling group's perspective. In North America, such structuring perspective was largely driven by white puritan conceptions of gender. The most recent changes have been more drastic however, as gender has taken on a less binary nature, reflected in the myriad new identifications (see e.g., the “54 Gender Identity Terms Every Ally Should Know” at https://www.refinery29.com/en-us/gender-identity-terms). In this section, we identify gender as a cultural script, and show how the structure of cultural scripts allowed gender to be fundamentally fluid.

Before we begin this discussion, we should note that all authors are of European descent from a middle class background and that two identify as genderqueer and neurodiverse. The perspective adopted in this article is mostly North American, and the examples used mostly concern this subcultural group. However, we believe that the mechanism at play is applicable to all cultures and positions, as it describes a mechanism that is anchored in enactive cognition, even if the details of these positions are subject to change.

### Gender as a Cultural Script

According to a still popular neuroreductionist account of gender, evolution gendered brains to solve the distinct recurrent survival problems faced by ancestral men and women in an environment of evolutionary adaptation (Baron-Cohen, 2003). On such views, gender is often construed as a static binary state people embody based on the sex they were assigned at birth (Geary, 2010; Österman and Björkqvist, 2018). Cultural studies, however, increasingly understand gender as neither binary nor static (Coney, 2015), a view supported both in psychology (Gabbard and Wilkinson, 1996) and sociology (Linstead and Brewis, 2004; Valocchi, 2005). On this account, gender is a interpersonal negotiation between individual agents, highly dependent on context and individual backgrounds; specifically, cultures offer individuals gendered scripts, which allow them the ability to predict each other's future actions, establishing roles and expectations for everyone involved (Goffman, 1978; Simon and Gagnon, 1986).

On the enactive-ecological view of scripts we defend, gender is the enacting of culturally gendered sensorimotor affordance loops and, but derivatively, the bodily and psychological consequences of enacting such loops. On this view, as we explain below, affordances landscapes are segregated human according to biological (i.e., Genetic-Gonadal-Genital or GGG sex—which is mostly binary (Fine, Joel REFS) sex in the hereronormative matrix. By setting up distinctive affordances landscapes for human males and females, specifically, by contrasting masculine to feminine, each pole has its own segregated set of associations (Rutter and Schwartz, 2011; Weisgram et al., 2011; Lee and Tang, 2015; Lindsey, 2015). Environments will afford actions to agents embodying one of the gendered poles, but not to agents embodying the other (Valenti and Gold, 1991; He and Zhou, 2018). Gendered public restrooms offer a simple example, identical in structure to the restaurant case described above. The entrance to public restrooms in a gendered culture is designed to provide an affordance landscape in which each door affords urinating, etc., to individuals assigned one gender but not to those assigned the other. As a result, individuals culturally assigned different genders will enact different sensorimotor affordance loops when the need comes to perform these basic physiological needs. Enacting culturally gendered sensorimotor affordance loops like the one provided by the public restroom affordance landscape is, in the first instance, what gender is all about. There are, of course, other elements constitutive of gender, we underline three here, to which we will refer back below. The first is a culturally gendered body that superposes itself on the individual's sexed (male, female, intersex) body, modifying its biological functioning to a certain extent (Fausto-Sterling, 2000). The second is a culturally gendered narrative (e.g., “I am the sort of person who goes to the Men's restroom,” “Going to the Lady's restroom is wrong,” etc.) which, once entrenched in an individual's narrative consciousness, forms the basis of the individual's (psychological) self, an internalized gender that gives psychological meaning to the gendered pronouns “his” and “her” (we will discuss non-binary pronouns below). The third is linked to one of the functions attributed to gender in a gendered society, that is, is the communication of a social identity through identifiable actions. Once again, these are the actions gender normative individuals will enact, given the action possibilities afforded by the gendered environment they find themselves in. But features, we claim, are thus consequences of enacting culturally gendered sensorimotor affordance loops. They are what happens to biological bodies and narrative consciousnesses in individuals enacting sensorimotor loops in a society that genders its affordances landscapes.

The public restroom example is simple in that it is manifestly gendered (with words or pictograms), manifestly binary (two doors, one for each culturally recognized gender), and the actions afforded are straightforward (they are basic physiological needs). Of course, in a gendered society, environments that, like public restrooms, provide affordances landscapes in which individuals assigned different genders can only or mostly enact the set of action culturally restricted to one gender multiply, but are usually less manifest than public restrooms. They nevertheless provided a basis for the full spectrum of gender in a society. The following provides a number of specific examples that fit the enactive “culturally gendered sensorimotor affordance loops” model sketched above quite well. Weisgram et al. (2011), for instance, show that gender expectations and narratives constrain the professional repertoire any given individual will consider. Those were in part constrained by the values that permeated the field considered, and its commonality with the values a gender was meant to embody. A similar finding shows that there are even more insidious barriers to employment in the intrinsic roles gender are expected to take on. Lee and Tang (2015) showed that caregiving was taken on by women, and tended to restrain their possibilities for employment. Such complicated interplays play a large role in gendered affordances. Rutter and Schwartz (2011), on their end, focus on sexuality, and the ways in which gender acts on an individual's sexual categorization shapes subsequent feelings of desire, arousal and subsequent sexual practices. Similarly to professional development, individuals focus on possibilities constrained by their gender position, and exist in the social matrix. They choose behaviors, and are monitored by other actors who validate their performance, or in contrast push back against a given behavior. Lindsey (2015), in her book on gender roles, also lays out how gender shapes employment and sexuality relations. She adds to this literature by showing the impact of religion and education in the binarizing of the concept. She lays out how social institutions act as a catalyst for the crystallization of these roles, and the constraint of affordances. Actions associated with either of those conceptual clusters will be afforded to agents embodying either of the gendered poles, and refused to the others. Specifically, the interaction of the individual will be constrained by the social environment based on its reading of the individual (Wiederman, 2005). Affordances are not simply understood by the agent embodying them, they are also mediated by other agents who relate to what they perceive of each other's gender. Alternatively, individuals are aware of gender expectations on them, and negotiate these expectations sometimes through subversion. Comunello et al. (2021) studied how gender was negotiated as a different landscape of affordance when it came to dating applications. They found that users walked a thin line of adhering to gendered scripts, and subverting them enough to fulfill their goals of getting a romantic partner. Thus, the individual integrates which possibilities are usually afforded by the environment as a function of a position the individual wishes to take, and this position makes salient specific options in the affordance landscape for the individual. Through this salience, individuals shape their affordance field. To return to the bathroom example, when a trans or gender non-conforming individual ponders which restroom they should go to, they instantly know they are supposed to choose one based on their gender identification and perception. Their appraisal of which they will choose is based on how well they consider their gender identity to match the proposed options, and the expected response from the environment should they choose either option. The individual is embedded in their surrounding and social group, knowing its norms, codes, and expecting its responses. In this sense, gender involves a set of affordance-action associations with ramifications into practice.

In what follows, we propose that enactive “culturally gendered sensorimotor affordance loops” model helps shed light on gendered differences. Our model resembles in many ways Butler (1985) performativity model of gender. Butler does not view performativity as entirely conscious, but it entails that agents will enact what they understand to convey an identity within socially bound constraints they have internalized. Gender, for Butler (1985), is enforced upon the individual by institutions and social agents who need to read it off the individual. Our enactive model follows this exact pattern with its sensorimotor cultural pre-reflectively felt gendered fields of affordances. Butler also proposes that an individual's performance has some possibility for reappropriation, and that agents have some agency in the way that their performance is expressed and socially understood. We can link this to the previously mentioned polysemic nature of scripts, and their capacity for change. Sensorimotor affordances-loops can change as a result of the actions of agents and their interaction with the world. By acting to change the structure of the world, for instance by removing the explicit gendered denomination of restrooms, one may sufficiently change the restroom affordances landscape to destabilize internalized sensorimotor loops. Finally, Butler proposes that individuals cannot simply escape social norms, because individuals need to remain intelligible (Butler, 1985). Even counter-discourse is thus subjected to the main discourse, as it directly hinges on this intelligible common discourse, and hence must define itself through opposition (Hird, 2000; Westbrook and Saperstein, 2015). This is directly relatable to scripts in the sense that scripts fashion our perception of the world and our understanding of social spaces. Scripts surround conceptual spaces and render the world intelligible (Levy and Fivush, 1993; Albarracin et al., 2020). In this sense, a gendered world is a world where interactions between gendered agents are predictable and testify to social intentions (Shapiro, 2015). By following these associations, agents also perceive the world through their own possibilities, based on their internalized positions (Izugbara et al., 2011). These affordances shape their effectual understanding of the world and of others around them. The performativity of gender is thus understood as the set of norm-regulated actions that individuals can undertake, actions that are determined by the cultural-affordance landscape generated by the previous action they undertook. This performativity is maintained by the cultural frame around the individual. Indeed, agents do not simply wake up with a choice (Butler, 1985). They are pushed and pulled in various directions by institutions and social representations (Dietert and Dentice, 2013). Agents are constantly reminded of the framework within which they operate, and their affordances are made salient based on the cultural codes at play (Bower and Gallagher, 2013; Heras-Escribano, 2016). Culture provides a segregated affordance landscape to males assigned at birth and females assigned at birth and gendered individuals perform the actions solicited by their normative affordance landscape (e.g., a picture on a door affords peeing to one gendered individual but not another) (Barrett et al., 2014; Gupta et al., 2019). More than simply proposing categorical separations, however, affordances are linked in terms of sequences. For instance, gendered restrooms are laid out differently and thus afford different actions for men and women (Staveren and Ode bode, 2007; Cislaghi and Heise, 2020).

Following Jackson (2006), the gendered segregation of affordances is not entirely arbitrary. They have a basis in a culturally determined sense of complementarity (Jackson, 2006), a structure that upholds the heteronormative framework (Geller, 2009; Janion, 2018). In that cultural frame, the sense of complementarity between men and women justifies that they need each other and are naturally drawn to each other (Varela et al., 2016). Genders are thus defined in relation to one another, as a sort of complementary all-encompassing whole (Hird, 2004). Feminine is defined against the masculine, with a different social valence associated to it (Keener et al., 2017). That is, masculinity is associated with more desirable qualities overall, although not all individuals are allowed to possess them (McCreary, 1994). This also serves to uphold the social hierarchy that places men above women. By reinforcing these narratives, affordances are offered to men in a way that they are refused to women. Scripts panning over lifetimes are drawn in this way with a clear sequence (Jordan-Young and Karkazis, 2019). White North American women were, for a long time, associated with care and home making, a situation that justified why they were not supposed to have a job or get away from their children for too long (Shome, 2011). Intersections of class and ethnicity could change this field of affordances by adding the opportunity to work in specific domains for women (Chisholm, 1972; Bell, 1992; Brush, 1999; Crenshaw and Bonis, 2005; Capodilupo and Kim, 2014).

Previously, we proposed that cultural scripts also define the roles individuals have to perform, socially or for themselves, and the interpersonal scripts to be enacted amongst one another. Specifically, cis-hetero-normative cultures would have males interact with females in a dominant manner, and females in a submissive manner (Herron et al., 1983; Buss, 1990). These larger life scripts embed smaller scale affordance-action sequences. For instance, white middle class women and sexuality are associated in contradictory manners, seen in a negative light because of stigmas surrounding sex work. Women whose actions afford sexuality are considered socially unacceptable since they threaten the social order of patrimony and legacy (Conrad, 2006). Hence, sexual unavailability is considered preferable. However, women are also objectified and portrayed as sexual objects of desire for men. Desirable women are preferable to sexually undesirable women, because the latter do not suit the tastes of men. This places women in the ambiguous position of only being able to receive sexual attention, but not to offer it, passively affording sexuality but denied actions that afford it (Conrad, 2006). This is a passive position. In contrast, white middle class men are considered better prototypes of their category if they can engage successfully in sexual conquest. This is an active position (Buss, 1990). This dichotomy entails a sort of natural sequencing where performing masculinity entails acting on initiating and pursuing affordances (e.g., a smiling woman affords initiating a sexually-directed social interaction), whereas performing femininity entails acting receiving and resisting affordances (e.g., a smiling man affords resisting a sexually-directed social interaction). In a typical seduction scenario, these opposing affordances have some ramifications about the sequence of events that may unfold. A man will possibly attempt to engage a relationship, and a woman will try to elude this relationship for as long it is still possible for her to do so without losing the man's desire. The asymmetric, and complementary, valence of the categories assigned to men and women directly impact the logical sequencing between the categories that become deployed into temporarily nested social scripts. These positions, based on categories to enact and associations to avoid, as well as their sequencing create identifiable roles for agents to embody. Roles are simply clusters of nested scripts that an individual can embody to guide their behavior in different settings in relation to socially sanctioned goals (Laner and Ventrone, 2000). A role acknowledges that individuals are positioned in the world and thus that it will not perceive affordances equally. Certain affordances, based on the script clusters the individual embodies, will be made more salient, and allow for normative social scenes to unfold predictably and relatively smoothly. If an individual, socially recognized through associations as signifying a role, fails to embody the scripts of that role according to social expectations, they are punished or ostracized by the group (Krane, 2018). Derogating from a role is risking to be socially unintelligible, and thus, unpredictable, affording “the wrong” actions gender wise. This lack of predictability can be bothersome for other agents for a host of reasons, including the generation of prediction errors, the breakdown of cultural sensorimotor loops, and the social threat to hierarchy and functioning (for detail see Krane, 2018; Albarracin et al., 2020). Certain individuals or groups have more to gain from the social roles being maintained (Harding, 2009; Pease, 2013). Those whose valence is high will probably benefit more from a given system being upheld, and may be more active in trying to maintain this social order (Flood and Pease, 2005; Kray et al., 2017). It is even possible that people who highly benefit from that system may not even perceive the issues it presents for other agents because their perceptual field is made of their own set of affordances, defined by the scripts they embody (Hardin et al., 2009).

### Sources of Fluidity in Gender

We suggested above that scripts are hybrid structures, part cognitive, part environmental that guide behaviors. Specifically, they are sequences of cultural affordance loops an agent acts on the pre-reflectively nested fields of affordances she perceives. We also saw that that scripts are not entirely fixed, devoid of the possibility for change, due in part to the polysemic nature of fields of affordances, and hence of scripts, and the relative ease which wich some fields of affordance can be culturally changed (as opposed to neurologically encoded sequences of action). Finally we saw that gender in a gendered society can be seen as sets of scripts, that is, sequences of loops made of gendered cultural affordances (e.g., gendered restrooms, work environment) and the normative response of individuals who perceive them. An individual's gender can be construed as the set of such loops they enact. In what follows, we show how our ecological-enactive model of gender described above can account for the sources of fluidity (this section) and we show this can explain the recent change and expansion of gender in some cultures (next section).

Giddens (1986) and Sewell (1992, 1998) explains that fluidity is possible because of the polysemy of scripts and resources. Scripts can mean different things to different people, or even to the same people in different scenarios. Given similar fields of affordances, two individuals may respond differently, and thus enact different scripts or may choose to perform a script differently. Script performance still needs to serve as an intelligible tool of social prediction and communication, and individuals negotiate their gender in relation to a sort of give and take. Individuals in a situation may wish to express a role they embody, and this role will be partly defined by what affordances other agents offer for the completion of this interaction.

Individuals are rooted in the specificity of their identities and experiences. Their environment is formative not only of their perception but of the available scripts at any given point. Since the interpretation and expression of scripts is dependent on the various groups an individual belongs and has been exposed to, position is an important determinant of scripts. Through this view, we understand that individuals will interpret their reality with a very specific schema, which is related to what they have come to experience and has made their conceptual space (Stoetzler and Yuval-Davis, 2002; Kronsell, 2005). As scripts require interpretation, and an environment, a specific perspective defines how a script will be interpreted, which then leads to the degrees of variance we observe. Subsequently, the different spaces an agent may be part of requires that they respond appropriately to the groups' expectations which will differ from group to group (de Vries, 2015; Atewologun, 2018).

The contextual expression of a script thus depends on the agents present and their respective understanding of those scripts. Social dynamics emerge thus, in a semi-predictable manner, when accounting for individual variability.

However, culture is an overarching structure, and a hierarchy still puts one format of femininity and masculinity at the top, referred to as hegemonic by the literature on gender (Connell, 2013). This is achieved through massively acquiesced (or imposed) narratives about ideals. Iconic and literary representations offer a stable and coherent picture of what constitutes the gendered ideal. This ideal is upheld by institutions and groups who stand the most to gain from it. For instance, women are pushed out of the STEM fields in droves, even when there were governmental efforts to push girls into STEM fields educational paths, in part because of stereotypes about women (Cheryan, 2012; Mangan, 2012; Dwyer, 2013; Hazelkorn, 2018). Concurrently, each gender is not only defined against its binary opposite, but also against itself. Femininity is not a unitary field of one prototype, and neither is masculinity. Feminine and masculine representations and interpretations measure against the hegemonic prototype, and cluster around different types of enactment (Paechter, 2003; Allan, 2009). This creates different prototypes, all hierarchically positioned. Different dynamics are made possible by this hierarchical variety of scripts, and the interactions between the two sides of the binary gender frames. This entails that each individual negotiates their social position in relation to other members around them and gets to renegotiate this position when different versions of the binary poles are presented.

Gender expression (or action from the perception-action pair, or Butlerian performance) is also renegotiated when other scripts come into play. For instance, it is not always advantageous to be a woman in a male-dominated industry, given personal goals and ranking in the industry (Corcoran-Nantes and Roberts, 1995; Wright, 2016). The same is not true in a gender equally distributed industry, or a female dominated industry (Gardiner and Tiggemann, 1999). Identity, in part, is communicative. It is not simply a reflection of the self, but an intelligible message that guides external expectations about the self. Through performance, this identity is made manifest as it is understood by the individual, and constrained by the group. There is a sense of agency in the negotiation of what one might wish to express to the external world. For instance, wishing to be seen as one identity marker over another may mean emphasizing one script and limiting the expression of another. While not all these negotiations are fully conscious, some are. Agency, in this regard, manifests over layers of consciousness, and constraining choices. Agency over scripts is thus a source of fluidity.

This hints at certain social dimensions which push individuals to evaluate their scripts, given the social expectations around their social category. Specifically, the intersection of certain scripts requires some reinterpretation in order to be maintained. If a woman in a male dominated industry wants to be read as feminine, all the while being read as a valid coworker, she must adopt a different interpretation of either of these scripts, which makes them intersect in a specific and complex way. The associated mental states involved in this reinterpretation may be explicitly conscious, but we venture to claim that in many cases they are pre-reflective, that is intuitively felt as the best thing to do in context. When there are available scripts, with sufficient overlap to be adapted, norms can be upheld. However, the social binary being as it is, it may not be sufficient to find overlap. There may not be overlap that is intelligible, or leads to acceptable social consequences for the individual. In that case, the individuals may have to create diverging scripts, offering a new pathway. This new pathway may require a community to become legible, stemming from populations for which the current framework simply does not work to account for their experience of reality. In turn, new identities emerge and become clusters with their own scripts, legible by more than the community they stemmed from. Even in the example above, where the scripts allowed the woman to navigate both identities, in time, and through meeting others such as her, she may consider that this intersection is a new integrated identity, with its own specific set of scripts. This generativity can span over various scales.

A script is not simply a means to communicate, but it is meant to enable coordinated action in social settings (Tison and Poirier, 2021a,b). Reading the complex group interactions, and the priors that are set by each group's expectations, an individual may interpret and manifest their social scripts in a way that is most beneficial for them in terms of utility, or least harmful for them in situations a power asymmetry.

Advantageous behaviors are thus mediated by the gender of the individual and the perception of that gender in relation to its coherence with the chosen role by the other members of the group. This is where the negotiated aspect of affordances becomes most evident. Two poles are important in this negotiation. The individual projecting their gender identity has a perception of themselves, and of what is expected of them. They are aware of how they will be read. Individuals tend to be aware of the valence that accompanies any particular reading. The second pole is the audience of the individual which has their own representations, and is likely to read what is projected by the individual. The emergent nature of the interaction is due to the position inhabited by the audience. Being in a gendered position is fundamentally being positioned in relation to others. The feminine position takes on a different nature when it is faced with the masculine position or the feminine position. Hence, taking on a feminine role with a masculine presenting person entails a series of roles and scripts, different than those expected if interacting with a feminine-presenting person. Given these expectations, different behaviors become more or less advantageous in order to achieve one's goals. There is, as a result, an intrinsic variability to gender expression and affordance landscape due to the variation of positionality embodied by the individual and their audience. This positionality is not simply defined by gender. Many other identifications can be embodied and read by the interlocutors, which mediate what expectations, and what roles they take on toward one another.

Thus, if class scripts and gender scripts intersect, their manifestation may be different. It may also be different when meshed with the manifold possible combinations of other social agents. Intersections of race may also change the way gender is defined and expressed, or even valued depending on the community where the agent is embedded (Gardiner and Tiggemann, 1999; Staples, 2005; Murphy et al., 2013). This entails a natural polysemy of gendered script, as well as a variety within those gendered scripts. Agents who can embody this variety have within their concepts a large array of clusters they can draw from at different times.

The very concept of discrete categories (binary genders) may be challenged by this polysemy, rooted at the heart of the complexity of an individual's specific position. Integrating cultural narratives into our measurement of differences and commonalities may help identify a finer grained analysis of phenomena.

Because social contexts overlap and interact, our semantic contents associated with any given script is filled and pulled by the ways these scripts are meaningful in these different areas. Being a neuro-diverse woman has a meaning that is different from being a white woman. Bringing these two clusters together creates an even different type of meaning. We can highlight how different dimensions offer very specific constraints over behaviors, based in part on the ways expectations shape how an individual will be interpreted.

Based on the way interpretations are placed on them, individuals have to interact with the world in very different ways. This is where the reinterpretation of scripts becomes central. All scripts are not necessarily perfectly suited for all intersections. Some intersections are more common than others, depending on how broad the category of identity is, and on how prevalent it is in society.

The previous examples also highlight the contextual reality of gender. Any given individual may be more advantaged from embodying one version of a script over another at any given time. Agents with a larger variety of available scripts have a greater capacity for adaptation to most situations. It is thus highly adaptive for agents to have a greater capacity for change in their repertoire and be less constrained while staying intelligible.

### Gender Has Changed and Expanded

Scripts have a form of porosity as well, due to their loosely coupled structure. The fluidity intrinsic to scripts is due to their polysemy, but agents integrating the scripts do not acquire them once, and forever hold them as they are. Agents can integrate new affordance and new associations. Clusters of associations can arise in social settings due to stable intersections for instance. The condition for a change cultural is that it can be picked up by other agents, thereby gain social communicability. Hence, when an agent can associate a set of behaviors to a concept they already have, their associations clusters expand. If we come back to the examples of the different manifestations of gender in terms of hierarchy, one agent can still recognize the gender expressed by another agent, even if it is not fully compliant with the social prototypes. Moreover, communities can form around specific manifestations of gender expression, and create a valence around this manifestation.

If in the general society system, new categories become more valent, different expressions of gender may become the new hegemony. Different sub-communities with different social attributes still value different types of femininity (Paechter, 2003, 2006; Valocchi, 2005; Schippers, 2007). Sub-communities can easily form and gather in urban settings because the density is higher and the probability of similar people finding each other follows. That is why urban settings are constituted of pockets of internal different valence that individuals may have to navigate over time (Nicholls, 2008). Such individuals are exposed to a variety of versions of scripts differently valued and must themselves adapt their own expression to each setting by integrating these versions to their concepts of the script in order to stay personally coherent. This promotes expansion of the script clusters for individuals exposed to this variety. In a context where this overlap happens, for any given individual, associations to gender roles become larger. For instance, in North-America, available behaviors for women went from being entirely segregated from masculine behaviors to overlapping strongly with most aspects of masculinity (Keener et al., 2017). There are still some elements of masculinity that are frowned upon for women in most cases, like the presence of faciale-hair. Hence the segregation of this aspect of scripts is still relatively defined.

Technology now allows people with similar conceptual clusters to find one another without having to be physically close (Code and Zaparyniuk, 2010). Communities can form and develop enough density that their script cluster develops an internal valence. The possibility for people with alternative models of sexuality can find each other this way. A good example of this is BDSM and LGBTQA communities developing online, and creating their own codes to communicate, as well as normalizing role models. These role models also create intelligible performances for members of the groups to be able to find one another and communicate. In the same way, versions of gender can gain some community traction, and turn into larger and more intelligible scripts by virtue of being adopted by multiple people to communicate with one another. Because individuals are exposed to a variety of sub-communities, and must negotiate gender in contact with them, our concepts of gender grow just as though they were physical communities (Satinsky and Green, 2016; Scaptura and Boyle, 2020).

All these variations have vastly contributed to the expansion of the masculine and feminine gender clusters of scripts to the point where they are not clearly separate anymore in some areas of subcultures where more personal freedom is afforded. Under the larger concept of masculinity can be found many scripts also suitable for the larger script of femininity. In this way, the concepts are not segregated anymore and the associations between the categories have expanded to largely overlap, they may not allow for easy segregation of the gendered patterns and ultimately for predictability (Richards et al., 2016). The gendered script may have reached a point of assimilation where it may not be possible to continue assimilating concepts usefully for all members of a larger North-American cultural group. Specifically, gender may have reached a point where it will either be entirely deconstructed or reorganized. This is not to say that gender norms are not enforced everywhere. There are still areas in the world, and cultural domains where gender norms are violently enforced (Bettcher, 2007; Wirtz et al., 2020). However, there are clusters where the gender construct is starting to lose meaning, especially in more urban middle class settings. This new overlap and expansion has given rise to a new accommodation of the gender scripts. As individuals are increasingly exposed to a variety of cultures, and mass media, opportunities to cross and inhabit different groups, settings and contexts increase.

New clusters and categories have developed into new possible identities. Previously unavailable identities that were simply unintelligible in terms of gendered scripts can now be read because they have garnered enough momentum and power to be socially meaningful. Similarly, the porosity of the gender concept has allowed agents to see themselves not as segregated and stable, but as fluid and possibly interchangeable.

As we have proposed seen, the gender script seems fluid, and likely to be polysemous even in a momentary stable interpretation. Hence, while individuals have to fluidly interpret their gender expression, they are also likely to be read in more fluid ways by others around them. The social group is comprised of people with different ways to interpret scripts (Goddard, 2009), and may make different gendered inferences because their certainty of a gender or another is low. This is especially true given the large overlap between the gender affordances. As this overlap widens, it becomes more evident that gender is not a direct correlate of sex, or even natural by most interpretations of that concept. By extricating gender from the natural sphere (whose change is generally considered slower than the cultural sphere), it becomes more easily understandable that gender may be subject to change for one individual. Observing more examples of gender expression that do not match the sex assigned at birth promotes the idea that gender is more performative than it is biological. Similarly, witnessing individuals who perform gender fluidly with skill also gives credence to the idea that an individual can easily embody gender expressions with fluidity when it suits their purposes. Dragqueens and Dragkings are perfect examples of this phenomenon. But more subtle examples are embodied by individuals who, as a function of context, go through different positions without necessarily embodying different genders. Some individuals have become more likely to accept that their gender identity does not have to directly match with the sex they were assigned at birth (Deutsch, 2016). This suggests that the categories around gender and naturality gained distance due to the loosening of the gendered segregations. In the same way, new possibilities for identifications that mix both masculine and feminine components have become possible and normalized. Bi-gender, androgyne or genderfluid individuals may change their expression from day to day, or consistently mix culturally clear elements of the binary script (BrckaLorenz et al., 2017). Alternatively, individuals have entirely reconstructed genders outside of the bounds of this initial binarity. People identifying as non-binary, aliagender, aporagender or agender have entirely come out of this conceptual boundary (Whyte et al., 2018; McCarthy et al., 2020).

While not entirely mainstream yet, these identifications are being adopted increasingly, mostly within the younger population, who by definition has been less exposed to more traditional gender roles (Richards et al., 2016). These gender identifications allow new script clusters and affordances for the people identifying with them, which is also one of the reasons to adopt them. Feeling like one's affordances represent how we feel, specifically, feeling like we can behave and present in a way that feels appropriate to ourselves is a specifically salient reason to choose a specific identity label (Weichold and Thonhauser, 2020). Choosing the label signals to other members of our community how we wish to be read. It normalizes the cluster we embody, and it allows individuals to reinforce their affordance field. Individual thus expect themselves, through this self-identification in a certain set of states, and act in order to tend toward those states. If the expectations are not shared by the group, this can lead to issues.

For instance, in areas where the gender binary is still very meaningful and enforced, individuals embodying more blurry boundaries whether through transition or non-binarity can face severe violence (White and Goldberg, 2006). This violence may go from non-intentional, such as being misgendered routinely without malicious intent, to the more nefarious intentional violence of actively punishing through physical violence a person for their gender expression. The transgender population is still one of the populations that faces the most risk of violence and, partly as a consequence, is also one of the populations with the highest rate of mental health issues due to minority stress (Hendricks and Testa, 2012).

Indeed, outside of the communities who can easily read these gender identifications, people adopting them may thus still face resistance, which means their affordance fields are complex interactions of their very own intersections and the way in which the environment will react and constrain them. Embodying their intersection may also prove troublesome in mainstream society since their bodies will be read with difficulty, or brought back into the binary folds, as non-prototypical expressions, and therefore lower on the social hierarchy. As individuals continue to embody these identifications, they will slowly influence gender expression possibilities, and their communities may grow to points where they become intelligible by an increasingly large portion of society. They will thus impact their niche, and push the conceptual clusters to continue evolving, and may in time create a system that allows for more options than binarity. As we move out of binarity, it is also possible that the possibilities for embodiment will change, and our entailed concepts of sex and sexuality may evolve as well.

## Conclusion

In conclusion, we very briefly review our view of gender, and almost just as briefly touch on some related issues from the gender literature. Genders, on the enactive-ecological view proposed here, are enacted by individuals as they come to perceive and normatively respond, that is, perform the action afforded, to the gendered landscapes of affordances built and maintained by their culture (especially by its institutions; or more generally by what Foucault calls its dispositif). In a binary gendered culture, clothing, restrooms, seats, school and college classes, offices, labs, streets, buses, smiles, greetings, gazes, usual phrases, emotional expressions, testimony, knowledge, etc., etc., afford different things to men and women. In such a culture, individuals become men or women as they come to pre-reflectively perceive the fields of affordances associated with one of the two genders and automatically normatively respond to them, setting off the chains of perception-action loops (gender scripts) through which a gender is enacted. As perception and automatic responding are both pre-reflective, enacting a gender is not a matter of conscious choice, though individuals can apply voluntary consciousness to resist, modulate, or otherwise modify an automatic response, opening a path toward a non (or less) normative gender for that culture.

On this view, gender is embodied since the affordances individuals can perceive depend on their action capacities (what an individual's body can and cannot do), which partly depend on its body (its anatomy and physiology). Biological sex, of course, is one part of an individual's anatomy and physiology, and as such affects its action capacities, enabling some, perhaps facilitating others. But on the view presented here, biological sex's link to gender is necessarily mediated by action capacities, which are dependent on the body as a whole and not just on its sexual characteristics. Biological sex may also be partly responsible for the affordances others perceive in an individual, especially in cultures that choose to amplify some of these differences, for instance those associated with secondary sexual characteristics. The same is true with body characteristics that don't have anything to do with biological sex (e.g., hair length). Since no two bodies and action capacities are alike, each gender, finely grained, is individual. There is no universal Man, and no universal Woman. And since bodies and actions capacities change throughout the lifespan of individuals, so does their gender at this fine grain. Of course, even if every single body is distinct, similarities abound, creating zones of density (distinct genders clustering around types) and zones of relative sparsity; like a starry sky filled with individual stars that cluster but also give rise to sparsely populated regions.

Gender on our view is also situated since the fields of affordances individuals can perceive depend on the affordances their culture provides, and affordances are (relational) properties of objects and situations in the individual's environment. Gender can only be enacted when individuals perceive the presence of gendered cultural fields affordances, and thus their enaction at a given moment is situated where such affordances fields are present; without gendered fields of affordances, there can be no gender. The actual fields of affordances culturally offered to individuals following an action simultaneously depend on all these factors. There is no such thing as a man or woman tout court because the affordances any individual can perceive in a situation are part of intersectional fields of affordances. The intersectional situatedness of genders is another source of gender variability and similarity in genders, as the fields in which individuals find themselves are always different, but share some similarities in various dimensions.

We saw that gender is also normative. It is normative in the first instance because, in the active inference framework we base our enactive-ecological view on, the presence of a field of affordances is not detected (and here we depart from strict Gibsonians) but predicted, a situation that generates a pre-reflective expectation of the field. When we wait at the entrance of a restaurant, we expect to be met and seated by someone from the restaurant staff. When this does not happen, the habitual flow of the restaurant script is disrupted and we must resort to conscious thinking to resolve the situation. These expectations set up norms for the smooth flow of action (and possibilities of voluntary disruption if one wishes to do so). The disruption of the smooth flow of action may be met with bemusement, or perhaps joy, by those one interacts with, but is often met with annoyance and sometimes violence. For a variety of reasons, individuals but also governments and other institutions may attempt to limit or ban some of these disruptions (e.g., in the fields of affordances associated with road circulation). Similarly with gender scripts, the failure of an expectation associated with a gendered cultural affordances field may disrupt the smooth flow of gender scripts, a situation that may be met with anything from bemusement to violence to legislation.

This is how gender is normative in a second instance: some individuals, governments and other institutions may have invested large resources to policing (sometimes violently) gender norms, a situation that may call for political action when genders or groups unjustly affected by such policing and violence must redress wrongs they suffer in the name of the smoothness of interpersonal action. Moreover, some individuals, governments and other institutions may have found it advantageous or expedient to use gender norms to set up and entrench political and economic power by controlling the types of fields of affordances offered to various genders and then strictly enforcing respect of the resulting norms.

This brings us to the last aspect of gender our account underlies, that is, the fact that gender is bound with justice issues. We said that the gendered cultural affordance landscape is built and maintained by a culture's institutions (or more generally its dispositif). This opens the question of distributive justice: are affordances distributed equitably in a given gendered society, or do they favor one gender over another economically, policially, etc., and, if they are not, what actions are available to ensure an equitable distribution of the fields of affordances.

## Author Contributions

MA devised the links between gender theory and enactive theory. PP worked more closely on the cognitive science aspects and laying out the foundations of theory used in the article. All authors contributed to the article and approved the submitted version.

## Conflict of Interest

The authors declare that the research was conducted in the absence of any commercial or financial relationships that could be construed as a potential conflict of interest.

## Publisher's Note

All claims expressed in this article are solely those of the authors and do not necessarily represent those of their affiliated organizations, or those of the publisher, the editors and the reviewers. Any product that may be evaluated in this article, or claim that may be made by its manufacturer, is not guaranteed or endorsed by the publisher.
